# An uncommon presentation of abdominal mass in an infant: A case report of fetus in fetu

**DOI:** 10.1016/j.radcr.2025.03.073

**Published:** 2025-04-17

**Authors:** Muhammad Idrees, Mushtaq Ahmed, Waleed Tariq, Muhammad Junaid Tahir, Anis ur Rehman

**Affiliations:** a Department of Orthopedis, Lahore General Hospital, Lahore, Pakistan; b Department of Pediatric Surgery, Sheikh Zayed Medical College, Rahim Yar Khan, Pakistan; c Department of Thoracic Surgery, Mayo Hospital, Lahore, Pakistan; d Department of Radiology, Shaukat Khanum Memorial Cancer Hospital and Research Centres, Lahore, Pakistan

**Keywords:** Fetus in Fetu (FIF), Contrast-enhanced computed tomography (CECT), Ultrasonography (USG), Magnetic resonance imaging (MRI), Alpha-fetoprotein (AFP), Beta-human chorionic gonadotropin (β-HCG)

## Abstract

Owing to the rare existence of fetus in fetu (FIF) with an incidence of one per 500,000 live births worldwide, embryologically it is characterized as diamniotic, monochorionic, monozygotic twins partially developing within the twin body. We present a case of a 10-month-old female suffering from progressively increasing abdominal distention and an immobile abdominal mass which was highlighted on contrast-enhanced computed tomography (CECT) consisting of soft tissue, gaseous, and fluid components. It was deciphered after surgical intervention as a stunted fetus characterized by the presence of structures such as the auricle, eye impressions, limb, vertebral column, brain, and gastrointestinal tissue. Such findings suggest the case of a fetus in fetu. Fetus in fetu must be considered as a differential diagnosis among infants and young children presenting with abdominal distention. Such a presentation must never be overlooked and probed clinically and radiologically, followed by surgical intervention to relieve the pressure effect over the viscera and abdomen.

## Introduction

German anatomist Meckel reported the first case of a fetus in fetu (FIF) in the early 19th century, a rare developmental anomaly with an estimated incidence of one in 500,000 live births worldwide. Embryologists hypothesized that it is secondary to a diamniotic, monochorionic, monozygotic twin pregnancy, in which an abnormal fetus gets encased within the body of a normal developing host [1]. This theory was evident by identical sex chromosomes, DNA and, protein polymorphisms, and blood grouping in FIF as in the co-existing twin. With more male predominance, its occurrence in the body was recorded more in the retroperitoneum than in the other body areas such as the cranial cavity, scrotum, and mesentery of the bowel wall [2].

The significant role of imaging modalities such as ultrasonography (USG), computed tomography (CT), and magnetic resonance imaging (MRI) in diagnosis and treatment cannot be ruled out. FIF must be used with a scoping view during early management [2]. We are reporting a rare case of FIF found in the left hypochondrium, which is attached to the mesentery of the gut and is not related to the retroperitoneum.

## Case description

A 10-month-old female presented with gradually increasing abdominal distension for the last 6 months which was associated with abdominal pain, excessive crying, sleeplessness, and fatigue and not associated with fever, vomiting, constipation, loose stools, jaundice, chest discomfort, cough, or sore throat. This infant was born through vaginal delivery at the gestational age of 38 weeks to a mother of 3 children, and she was married contagiously. On further inquiry, the antenatal history of the patient and maternal history of exposure to radiation, drugs, or any chronic disease were insignificant. The family history of congenital anomalies and twin pregnancies was unremarkable. She achieved developmental milestones according to her age until presentation. Her parents consulted various physicians during this period with no satisfactory relief, and she was managed with supportive therapy (i.e., fluids, multivitamins, and analgesics).

On examination, a cystic, nontender, immobile central abdominal mass of 12 × 10 cm with an elliptical shape and smooth contour was found encroaching the epigastrium and left lumber region. It was nonadherent to the skin, and the overlying skin was normal.

**Fig. 1:**
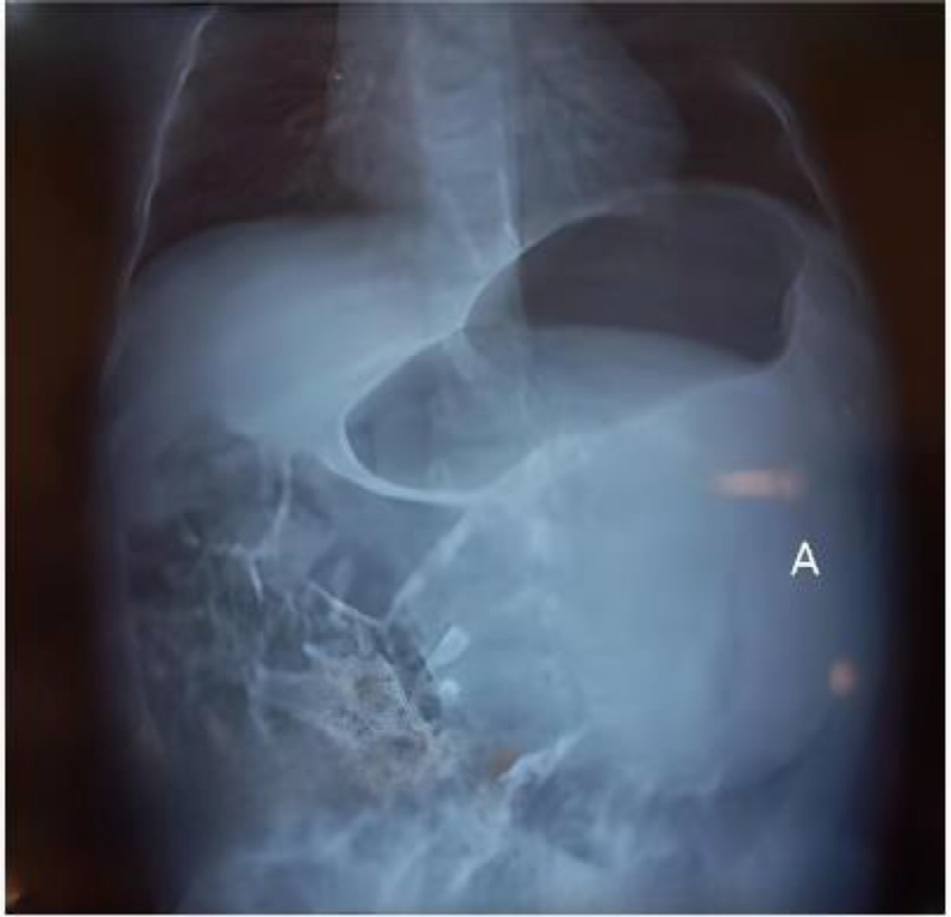
Frontal X-ray of a skeletal immature child. (A) demonstrating a predominantly cystic mass in the left hemi-abdomen.

**Fig. 2:**
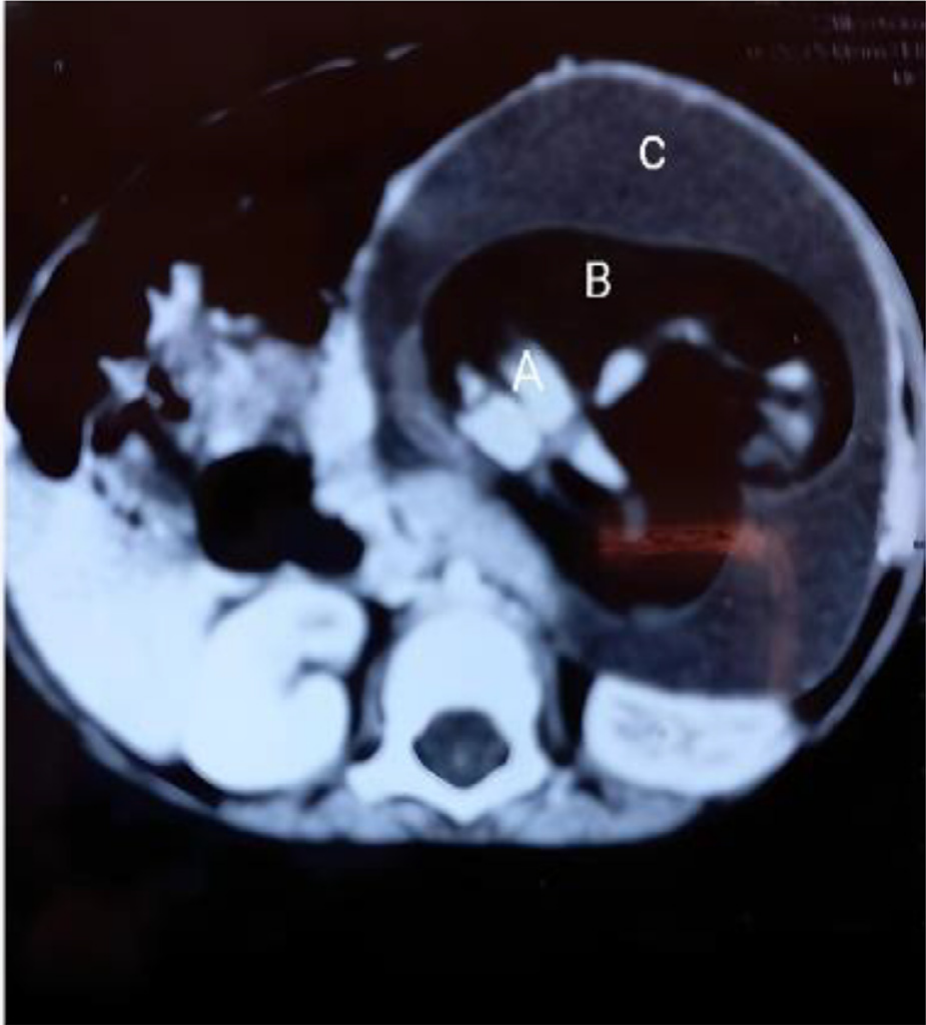
Axial view of CT scan showing a cystic structure in the abdomen (A: Soft tissue component, B: Gaseous component, C: Cystic fluid component).

**Fig. 3:**
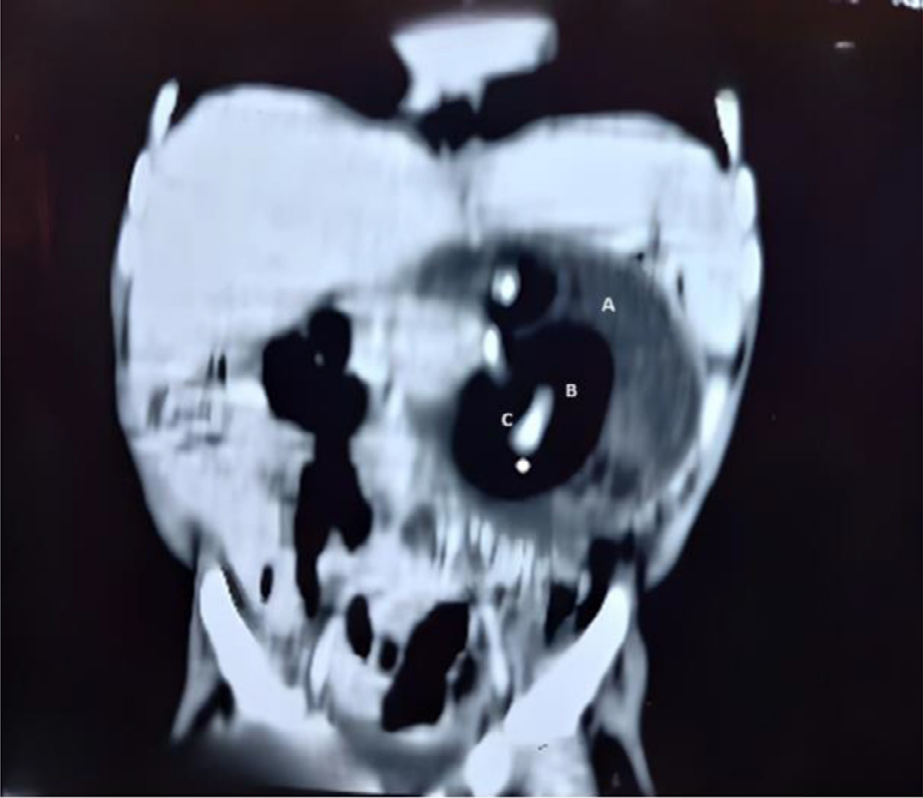
Coronal view of CT scan showing A (well-circumscribed heterogenous density mass in left mid abdomen and left lumbar region of 9.2 × 7.6 cm with smooth and sharp outline, having significant cystic (A), fat (B), and calcified component (C).

Radiographic findings suggested a soft tissue density mass in the left upper quadrant, imposing a mass effect with superior stomach displacement (Fig. 1). On abdominal ultrasound, a cystic mass of 12.1 × 11.2 × 10.5 cm (volume = 815 ml) with solid internal components was seen in the epigastrium and left hypochondrium region and was not tacky with the kidney or spleen. Contrast-enhanced computed tomography (CECT) depicted a well-defined oval-shaped mass on the left side of the abdomen, causing a mass effect over the left kidney, displacing it posteriorly and crossing the midline with the displacement of gut loops towards the right side. The mass showed soft tissue, gaseous, and fluid components with no significant internal vascularity (Figs. 2 and 3). Differential diagnosis of the mesenteric cyst or cystic teratoma was made after the initial diagnostic workup and surgical excision was planned as definitive treatment. The patient was prepared for exploratory laparotomy after fulfilling requirements like blood investigations and informed consent with detailed counseling.

**Fig. 4:**
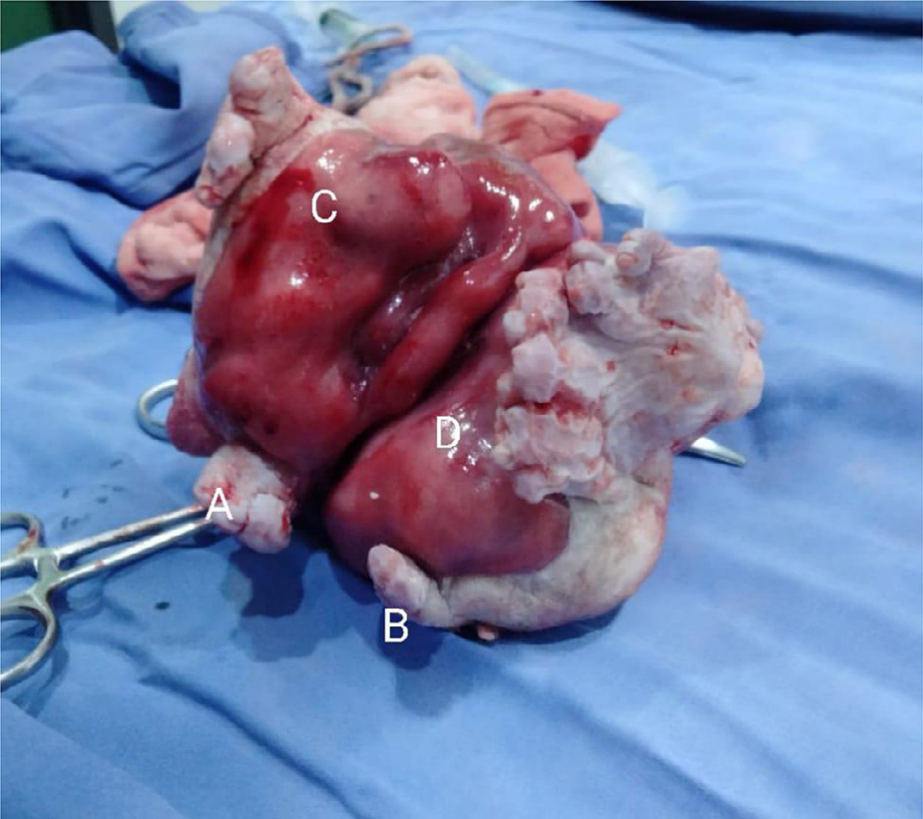
Gross specimen having features consistent with 9th week of development (A: Developing arm, B: Developing leg, C: Primordial eye, D: Developing heart**)**.

Elective laparotomy with a right upper transverse incision was performed. Intraoperatively, an enlarged cystic mass was found in the left upper quadrant of the abdomen, displacing the stomach and duodenum superiorly. The mass was attached to the mesentery of the duodenum with no retroperitoneal extension. The cyst was decompressed and excised along with a rudimentary blood supply from one branch of the superior mesenteric artery. On gross examination of the specimen, the stunted fetus of approximately 9 weeks was found with smooth skin covering the entire body from head to toe. Well-developed symmetrical auricles, eye impressions, and facial features were also observed (Fig. 4).

## Discussion

Ventral folding of the tri-laminar embryonic disc consisting of exoderm, mesoderm, and endoderm usually occurs during the 2nd and 3rd weeks of embryogenesis. The pathogenesis of a fetus in fetu was described as a growing monozygotic diamniotic monochorionic twin inside its host's body during 2nd and 3rd weeks of embryogenesis. Owing to the abnormal partition of the totipotent cellular mass, the developing embryo incorporates its small part within the sister larger one, documented as the fetus in fetu in the literature. The persistent anastomosis of the embryo's vitelline circulation was also a significant factor in its parasitic incorporation within the sister twin [3]. In our case, we found a small, stunted mass encaged within a live child, suggesting that it is secondary to a disrupted vascular supply.

Only 200 cases have been reported since the 19th century, with an incidence of one in 5 lacs live births over the globe [3]. Between 2008 and 2021, ten cases have been reported in India [1]. Such cases have also been documented in different hospitals in Pakistan, such as Agha Khan Hospital, Karachi [4], PIMS Hospital, Islamabad [5], and Children Hospital, Lahore [6]. However, data on the overall statistics are not available. We discuss our current report from a tertiary care hospital in Sadiq Abad, a small, populated district of Punjab, Pakistan.

Most cases (89%) usually present with abdominal distention and mass up to 18 months, with a maximum age of late presentation at 47 years [7]. The chief complaint in this case was also abdominal distention, which was reported at ten months, congruent with most reported cases. The fetus in fetu was commonly found retroperitoneally (80%), with a higher prevalence in males (63%), but other rare sites such as the cranial cavity, scrotum, lungs, hypochondrium, pelvis, and sacrococcygeal regions have also been reported [1]. In our case, it was a female infant with FIF found in the left hypochondrium, which falls in a rare category. Regarding its number, it could be one or more with a maximum number of 11 expressed in the data [7], while in our case, there was only 1 FIF.

The chief complaint of abdominal distention with mass raises suspicion of teratoma in infants, which usually occurs secondary to uncontrolled growth of embryonal stem cells with no definite organization. The presence of an axial skeleton in the form of a vertebral column with limbs aligned across it was hypothesized to be pathognomonic of FIF and a strongly extricated teratoma [8]. We followed the same clinical diagnosis tract and found a well-developed vertebral column in the current case. The vertebral column (76%), gastrointestinal system (61%), central nervous system (55%), genitourinary system (36%), and various other primitive organs were identified in different FIF specimens [9]. Our report recognized the vertebral column, limbs, brain, eyes, ears, and developing gastrointestinal tract.

FIF can be diagnosed radiologically using an initial radiograph and ultrasound. The presence of a vertebral column along with bony segments surrounded by soft tissue density on abdominal radiography suggested FIF, as in our scenario. However, the absence of a vertebral column never ruled out FIF, as a similar case was reported by Knox et al. without visualization of calcifications [10]. Heterogeneous masses with bony tissues on ultrasonography strongly supported FIF Additionally, CECT and MRI could be used to obtain more information regarding the surrounding structures and relationships, which might be helpful in surgical treatment [9]. We dealt with this case by diagnosing it via plain abdominal X-ray and ultrasound while planning CECT for the surgical approach. After resection, histopathology of the specimen could not be performed, which was attributed to a lack of financial resources.

The definitive treatment for FIF is complete and safe surgical excision. FIF is a benign disease that requires surgical intervention to manage its pressure effects. However, Hopkins et al.reported a single case of malignant recurrence of FIF attributed to incomplete removal of the adherent membranes at the surgical site. Postoperative surveillance and regular monitoring of serum alpha-fetoprotein (AFP) and beta-human chorionic gonadotropin (β-HCG) levels are advised at intervals of one month for one year. Furthermore, exhaustive histopathology is also advised to assess for any malignancy [11]. However, in our case, there was a loss of follow-up as a result, the patient could not be assessed for any morbidities.

## Conclusion

Recent advancements emphasized early diagnosis and management of FIF. Regular antenatal visits and sonography can detect such anomalies before birth. The presentation of a patient with recurrent complaints must always be addressed and be facilitated with expertise immediately. Complete safe surgical excision followed by histopathology should be preferred. Postoperatively, FIF must be screened with frequent follow-up visits and tumor markers, i.e., alpha-fetoprotein.

## Patient consent

The consent was acquired from the Guardian of the patient.
